# Transesophageal echocardiography for cardiovascular risk estimation in patients with sepsis and new-onset atrial fibrillation: a multicenter prospective pilot study

**DOI:** 10.1186/s13613-021-00934-1

**Published:** 2021-10-18

**Authors:** Vincent Labbé, Stephane Ederhy, Nathanael Lapidus, Jérémie Joffre, Keyvan Razazi, Laurent Laine, Oumar Sy, Sebastian Voicu, Frank Chemouni, Nadia Aissaoui, Roland Smonig, Denis Doyen, Fabrice Carrat, Guillaume Voiriot, Armand Mekontso-Dessap, Ariel Cohen, Muriel Fartoukh, for the FAST Study Group, Bertrand Guidet, Hafid Ait-Oufella, Simon Bourcier, Daniel Da Silva, Sebastien Jochmans, Jean Dellamonica, Jean-François Timsit, Bruno Megarbane, Jean-Luc Diehl, Sophie Rushton-Smith

**Affiliations:** 1 Sorbonne Université, Assistance Publique-Hôpitaux de Paris (AP-HP), Service de Médecine Intensive Réanimation, Département Médico-Universitaire APPROCHES, Hôpital Tenon, 4 rue de la Chine, 75020 Paris, France; 2 Université Paris Est, Groupe de Recherche Clinique GR05 CARMAS, Institut Mondor de recherche biomédicale, INSERM, Créteil, France; 3 Department of Cardiology, UNICO Cardio-Oncology Program, Hôpital Saint-Antoine, AP-HP, Paris, France; 4 INSERM U 856, Paris, France; 5 Sorbonne Université, INSERM, Institut Pierre Louis d’Epidémiologie et de Santé Publique IPLESP, AP-HP, Paris, France; 6 Sorbonne Université, Public Health Department, Saint Antoine Hospital, AP-HP, Paris, France; 7 Service de Médecine Intensive Réanimation, Hôpital Saint-Antoine, AP-HP, Sorbonne Université, Paris, France; 8 Service de Médecine Intensive Réanimation, Département Médico-Universitaire Médecine, Hôpitaux Universitaires Henri Mondor-Albert Chenevier, AP-HP, Créteil, France; 9 Service de Médecine Intensive Réanimation, Centre Hospitalier de Saint-Denis, Saint Denis, France; 10 Service de Médecine Intensive Réanimation, Groupe Hospitalier Sud Ile-de-France, Centre Hospitalier Melun, Melun, France; 11 Service de Réanimation Médicale et Toxicologique, Hôpital Lariboisière, AP-HP, INSERM UMRS-1144, Université de Paris, Paris, France; 12 Service de Médecine Intensive Réanimation, Gustave Roussy, Villejuif, France; 13 Service de Médecine Intensive Réanimation, Hôpital Européen Georges-Pompidou, AP-HP, Université Paris-Descartes, Paris, France; 14 Department of Intensive Care Medicine and Infectious Diseases, Bichat-Claude Bernard University Hospital, AP-HP, Paris, France; 15 Service de Médecine Intensive Réanimation, Hôpital l’Archet 1, Centre Hospitalier Universitaire de Nice, and UR2CA Unité de Recherche Clinique Côte d’Azur, Université Côte d’Azur, Nice, France; 16 UMR-S ICAN 1166, Sorbonne Université, Paris, France

**Keywords:** Atrial fibrillation, Transesophageal echocardiography, Stroke, Bleeding, Sepsis

## Abstract

**Background:**

Echocardiographic parameters have been poorly investigated for estimating cardiovascular risk in patients with sepsis and new-onset atrial fibrillation. We aim to assess the prevalence of transesophageal echocardiographic abnormalities and their relationship with cardiovascular events in mechanically ventilated patients with sepsis and new-onset atrial fibrillation.

**Methods:**

In this prospective multicenter pilot study, left atrial/left atrial appendage (LA/LAA) dysfunction, severe aortic atheroma, and left ventricular systolic dysfunction were assessed using an initial transesophageal echocardiographic study, which was repeated after 48–72 h to detect LA/LAA thrombus formation. The study outcome was a composite of cardiovascular events at day 28, including arterial thromboembolic events (ischemic stroke, non-cerebrovascular arterial thromboembolism, LA/LAA thrombus), major bleeding, and all-cause death.

**Results:**

The study population comprised 94 patients (septic shock 63%; 35% women; median age 69 years). LA/LAA dysfunction, severe aortic atheroma, and left ventricular systolic dysfunction were detected in 17 (19%), 22 (24%), and 27 (29%) patients, respectively. At day 28, the incidence of cardiovascular events was 46% (95% confidence interval [CI]: 35 to 56). Arterial thromboembolic events and major bleeding occurred in 7 (7%) patients (5 ischemic strokes, 1 non-cerebrovascular arterial thromboembolism, 2 left atrial appendage thrombi) and 18 (19%) patients, respectively. At day 28, 27 patients (29%) died. Septic shock (hazard ratio [HR]: 2.36; 95% CI 1.06 to 5.29) and left ventricular systolic dysfunction (HR: 2.06; 95% CI 1.05 to 4.05) were independently associated with cardiovascular events.

**Conclusions:**

Transesophageal echocardiographic abnormalities are common in mechanically ventilated patients with sepsis and new-onset atrial fibrillation, but only left ventricular systolic dysfunction was associated with cardiovascular events at day 28.

**Supplementary Information:**

The online version contains supplementary material available at 10.1186/s13613-021-00934-1.

## Background

New-onset atrial fibrillation (NOAF) is the commonest arrhythmia in the intensive care unit (ICU) occurring in one-third of critically ill patients with sepsis [1, 2]. In this setting, patients with NOAF are at greater risk of arterial thromboembolic events [2] and death than patients without NOAF [1, 2]. Decision-making with regard to thrombo-prophylaxis should be based upon the absolute risks of cardiovascular events, including arterial thromboembolism event, bleeding, and death, and the net clinical benefit for a given patient. However, conventional score to assess thromboembolic and bleeding risk in patient with atrial fibrillation have not been validated in the specific setting of sepsis [3, 4].

Transesophageal echocardiography (TEE) abnormalities, such as left ventricular (LV) systolic dysfunction, left atrial/left atrial appendage (LA/LAA) dysfunction, and severe aortic atheroma, are associated with an increased risk of arterial thromboembolic event in patients with atrial fibrillation [5]. In particular, LA/LAA dysfunction, revealed by LAA low velocities and dense spontaneous echo contrast (SEC) resulting from blood stasis [6–8], remains strongly associated with thrombus formation [9]. So, bedside echocardiography could be useful in critically ill patients with sepsis and NOAF for estimating their cardiovascular risk.

We therefore conducted a prospective, multicenter, observational, echocardiographic pilot study (the Fibrillation Atrial Sepsis Thrombus [FAST] study) in 10 French ICUs in patients with sepsis and NOAF. Our objectives were to assess early LV systolic dysfunction, LA/LAA dysfunction, and severe aortic atheroma, and to investigate the relationship of these structural and functional parameters with the occurrence of cardiovascular events at day 28.

## Methods

### Patient selection

During a 24-month period (November 2014 to November 2016), all mechanically ventilated adult patients with sepsis/septic shock who experienced significant NOAF (including atrial fibrillation and atrial flutter) on ICU admission or during their ICU stay were eligible. Significant NOAF was defined by an AF in patients with no prior history of AF [10], lasting at least 6 h or recurred more than twice (> 30 s) per day despite correction of modifiable risk factors such as hypokalemia or hypovolemia [11]. Sepsis/septic shock was defined according to the Sepsis-3 definition [12]. All episodes of NOAF were systematically recorded on an electrocardiogram assessed by two cardiologists (any discrepancy being solved by consensus), and classified as atrial fibrillation, or atrial flutter using standard definitions [13]. Patients with a history of atrial fibrillation for which the cardiovascular risk depends in part to their established previous medication regimen for atrial fibrillation [10], with valvular heart disease classifying AF as “valvular AF” (significant mitral stenosis, mechanical aortic or mitral valve; [14]), with TEE contraindication [15], those who were moribund (expected survival < 48 h), or with a decision to limit full care, and those refusing to participate, were not included.

The study was approved by the Comité de Protection des Personnes Ile-de-France 5 (ref. 14941), as a component of standard care, and patient consent was waived, as per French Law. Written and oral information about the study was given to the patients or their next of kin.

### Data collection

Demographics, medical history, antithrombotic medications, admission category, Simplified Acute Physiology Score II [16], and CHA_2_DS_2_-VASc [14] and HAS-BLED [14] scores were recorded on ICU admission (detailed definitions in Additional file 1: Table S1). The type of infection and the Sepsis-Related Organ Failure assessment score [17] were recorded at NOAF onset (day 0). NOAF management during ICU stay was left at the discretion of the physicians in charge.

### Echocardiography

Echocardiography were performed at the bedside by skilled intensivists, all of whom had ≥ 2 years of TEE experience, with competence in advanced critical care echocardiography [18]. Examinations were conducted with recent commercially available equipment (CX50, AFFINITY 70, and IE33; Philips Ultrasound system, Bothell, WA; VIVID 7, VIVID 9, and VIVID I, General Electric Healthcare system, Horten, Norway). All echocardiographic studies involved transthoracic echocardiography according to the recommendations of the European Association of Cardiovascular Imaging [19] with 2.5- and 3-MHz transducers, followed by TEE with 5-MHz multiplane transducers. Each patient underwent echocardiography within 48 h of NOAF onset (initial TEE). Based on the initial TEE findings, changes in treatment regarding therapeutic anticoagulation were left at the discretion of the physicians in charge. To investigate LA/LAA thrombus formation, a second echocardiography was performed 48 to 72 h after the initial TEE in patients who were still mechanically ventilated (second TEE). Supplementary echocardiography studies performed at the discretion of the physicians in charge were recorded.

LA or LAA thrombi, LA/LAA dysfunction (including LA/LAA dense SEC and LAA low velocities), LAA large area, and severe aortic atheroma were investigated as previously described [6, 7, 20–24]. A LA/LAA thrombus was considered present when there was a well-circumscribed, echodense, intra-cavitary mass that is acoustically distinct from the underlying endocardium and the pectinate muscles [21]. LA/LAA dense SEC was defined as finely reticular pattern of dynamic, swirling intra-cavitary echoes localized within the LA or LAA persistent throughout the LA–LAA at normal gain [6, 21]. Velocity of the LAA was recorded with pulse-wave Doppler interrogation 1 cm within the orifice. Flow velocity was evaluated in anterograde (emptying) and retrograde (filling) directions, and was averaged over a minimum of 3 to 5 consecutive cardiac cycles as specified by protocol. LAA low velocity was defined as a LAA filling or emptying velocity < 25 cm/s [7, 23]. The area of the LAA was measured by planimetry at 90° view as specified by protocol. LAA large area was defined as a LAA area > 5 cm^2^ [21]. The thoracic aorta was analyzed at each level, including the ascending aorta, the proximal and distal arch, and the descending aorta, as specified by protocol and according to previously described methods [20]. Severe aortic atheroma was defined as: protruding atherosclerotic plaques (highly echogenic areas that protruded ≥ 4 mm above the surface of the intima into the aortic lumen); or mobile atheroma; or ulcerated atheroma [20, 22, 24]. LV systolic dysfunction was defined as LV ejection fraction ≤ 45% (mild, 31–45%; severe, ≤ 30%) using the biplane Simpson method [19].

All echocardiographic studies were analyzed off-line by two experienced observers (VL and SE) in a blinded manner. Differences between observers were resolved by consensus; if the observers could not agree, a third observer (AC) reviewed the studies, and that observer’s judgment was binding.

### Follow-up and outcomes

All patients were followed from day 0 (inclusion) to day 28. The primary outcome was the occurrence of a cardiovascular event, comprising arterial thromboembolic events (ischemic stroke, non-cerebrovascular thromboembolism, or thrombus of the LA/LAA on echocardiography studies [3]), major bleeding event (according to the International Society on Thrombosis and Hemostasis definition: symptomatic bleeding in a critical area or organ such as intracranial, bleeding associated with a reduction in hemoglobin of ≥ 1.24 mmol/L or leading to transfusion of ≥ 2 units blood or packed cells, or fatal bleeding; [25, 26]), and death from any cause. Secondary outcomes were individual components of the primary outcome (detailed definitions in Additional file 1: Table S1).

With the exception of the second TEE, no systematic screening of arterial thromboembolic events was performed. The diagnostic work-up of arterial thromboembolic events and major bleeding events included the usual investigations performed in the participating ICUs, and was collected by the attending intensivists. An independent committee adjudicated arterial thromboembolic events and major bleeding events, and assessed the relationship of ischemic stroke and non-cerebrovascular thromboembolism with NOAF, using the Stop Stroke Study Trial of Org 10172 in Acute Stroke Treatment classification (detailed definitions in Additional file 1: Table S2, Fig. S1, [27]).

### Statistical analysis

Data are reported as medians (interquartile range [IQR]) for quantitative variables, and as frequencies (percentages) for categorical variables. Associations with composite primary outcome (cardiovascular events) were tested by standard Cox models. A multivariable model for the primary outcome was built from studied TEE parameters (LV systolic dysfunction, LA/LAA dysfunction, severe aortic atheroma), with additional adjustment on variables associated with this outcome in the univariate analysis. Associations with secondary outcomes (individual component of the composite primary outcome) were tested by univariate cause-specific Cox models for the first occurrence of arterial thromboembolic event or major bleeding event (accounting for the competing risk of death), and by standard Cox models for the all-cause mortality. A multivariable model was built for the primary outcome only, as the number of events was judged too low to avoid overfitting for the other outcomes. All survival models were censored at day 28. Hazard ratios (HRs) were estimated and reported with their 95% confidence intervals (CIs). All tests were 2-tailed and *p* values < 0.05 were considered significant. Statistical analysis was conducted with R version 3.6.3 (R Core Team 2019; R foundation for statistical Computing, Vienna, Austria).

## Results

### Baseline clinical characteristics and TEE findings

**Fig. 1:**
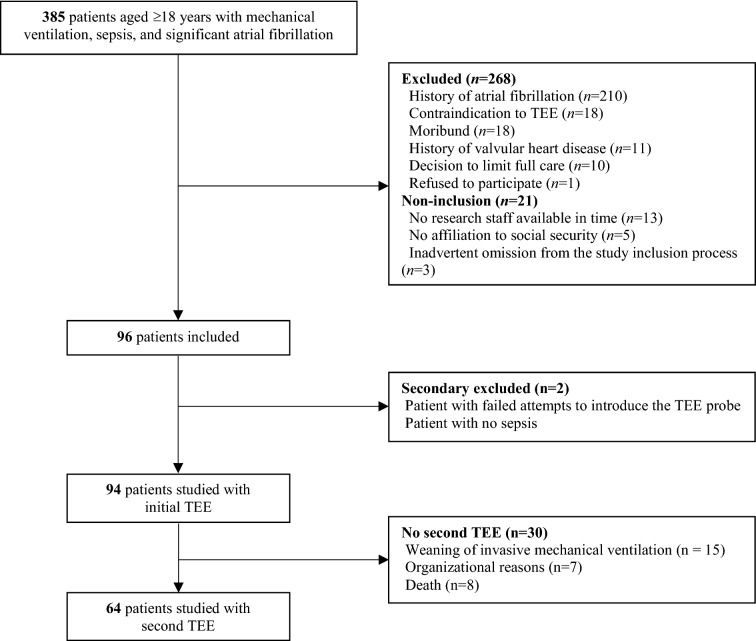
Patient flowchart. *TEE* transesophageal echocardiography

During the study period, 94 patients were studied (63 men and 31 women) with a median age of 69 years (IQR: 61 to 77 years) (study flowchart in Fig. 1). Median time from ICU admission to NOAF onset was 2 days (IQR: 1 to 4 days) (atrial fibrillation, *n* = 93; atrial flutter, *n* = 1). Baseline clinical characteristics are displayed in Table 1. Median CHA_2_DS_2_-VASc score was 3 (IQR: 2 to 4), and HAS-BLED score was 2 (IQR: 1 to 3). Initial TEE studies were performed after a median time of 1.3 days (IQR: 0.7 to 2.1 days) from NOAF onset. NOAF was present during initial TEE in 47% of patients (details regarding other hemodynamic parameters during TEE are available in Additional file 1: Table S3). LA/LAA dysfunction was detected in 19% of patients, including LA/LAA SEC (7%) and LAA low velocities (12%). Severe aortic atheroma was diagnosed in 24% of patients. LV systolic dysfunction occurred in 29% of patients. None of the patients had thrombus in the LA or LAA (Table 2). Regarding the therapeutic impact of TEE studies, therapeutic anticoagulation was initiated following TEE in 3% of patients due to LA/LAA dense SEC (*n* = 2) and severe aortic atheroma (*n* = 1).

**Table 1:** Baseline clinical characteristics, initial severity, and initial new-onset atrial fibrillation management according to 28-day arterial thromboembolic events, major bleeding events and death

Variable	Total (*n* = 94)	Arterial thromboembolic event		Major bleeding event		Death	
		No (*n* = 87)	Yes (*n* = 7)	No (*n* = 76)	Yes (*n* = 18)	No (*n* = 67)	Yes (*n* = 27)
***Baseline clinical characteristics on ICU admission***							
Age, median (IQR), years	69 (61–77)	69 (61–77)	69 (63–80)	69 (61–78)	67 (61–74)	69 (61–77)	68 (64–78)
Female sex, no. (%)	33 (35)	31 (36)	2 (29)	26 (34)	7 (39)	25 (37)	8 (30)
Cardiac disease, no. (%)	17 (18)	14 (16)	3 (43)	14 (18)	3 (17)	13 (19)	4 (15)
Left ventricular systolic dysfunction, no. (%)	3 (3)	3 (3)	0	3 (4)	0	3 (4)	0
Vascular disease, no. (%)	19 (20)	16 (18)	3 (43)	14 (18)	5 (28)	11 (16)	8 (30)
Stroke, no. (%)	8 (8)	8 (9)	0	7 (9)	1 (6)	5 (7)	3 (11)
Diabetes mellitus, no. (%)	24 (25)	23 (26)	1 (14)	21 (28)	3 (17)	14 (21)	10 (37)
Smoker, no. (%)	51 (54)	48 (55)	3 (43)	42 (55)	9 (50)	37 (55)	14 (52)
Hypertension, no. (%)	57 (61)	51 (59)	6 (86)	46 (60)	11 (61)	38 (57)	19 (70)
Clinical risk scores, median (IQR)							
CHA_2_DS_2_-VASc	3 (2–4)	3 (2–4)	3 (1–4)	3 (2–4)	3 (2–4)	3 (2–4)	3 (2–4)
HAS-BLED	2 (1–3)	2 (1–3)	2 (1–3)	2 (1–3)	2 (1–3)	2 (1– 3)	2 (1–3)
Previous therapeutic anticoagulation, no. (%)	4 (4)	4 (5)	0	2 (3)	2 (11)	4 (6)	0
Admission category, no. (%)							
Medical	74 (79)	70 (80)	4 (57)	61 (80)	13 (72)	52 (78)	22 (81)
Scheduled surgery	5 (5)	5 (6)	0	4 (5)	1 (6)	4 (6)	1 (4)
Emergency surgery	15 (16)	12 (14)	3 (43)^a^	11 (14)	4 (22)	11 (16)	4 (15)
SAPS II score on ICU admission, median (IQR)	58 (47–74)	58 (44–74)	63 (51–68)	60 (44–74)	54 (50–73)	54 (43–73)	65 (55–74)
***Severity and management on the day of NOAF onset***							
SOFA score, median (IQR)	9 (7–11)	9 (7–11)	9 (6–9)	9 (7–11)	9 (6–11)	9 (7–11)	10 (6–13)
Septic shock, no. (%)	59 (63)	55 (63)	4 (57)	45 (59)	14 (78)	38 (57)	21 (78)
Catecholamine, no. (%)	75 (80)	70 (80)	5 (71)	60 (79)	15 (83)	52 (78)	23 (85)
Norepinephrine	71 (75)	66 (76)	5 (71)	57 (75)	14 (78)	50 (75)	21 (78)
Epinephrine	6 (6)	5 (6)	1 (14)	3 (4)	3 (17)^b^	4 (6)	2 (7)
Dobutamine	7 (7)	5 (6)	2 (29)	7 (9)	0	5 (7)	2 (7)
Cardioversion attempt, no. (%)	60 (64)	58 (67)	2 (29)	49 (64)	11 (61)	42 (63)	18 (67)
Medical (amiodarone)	59 (63)	57 (65)	2 (29)	48 (63)	11 (61)	41 (61)	18 (67)
Electrical	16 (17)	16 (18)	0	9 (12)	7 (39)^b^	10 (15)	6 (22)
Antiplatelet therapy, no. (%)	24 (25)	21 (24)	3 (43)	17 (22)	7 (39)	15 (22)	9 (33)
Therapeutic anticoagulation, no. (%)	28 (30)	25 (29)	3 (43)	23 (30)	5 (28)	23 (34)	5 (18)

*CHA*_*2*_*DS*_*2*_*-VASc* congestive heart failure, hypertension, age ≥ 75 years (doubled), diabetes mellitus, prior stroke or transient ischemic attack or thromboembolism (doubled), vascular disease, age 65 to 74 years, sex category (female), *HAS-BLED* hypertension, abnormal renal/liver function, stroke, bleeding history or predisposition, labile international normalized ratio, elderly, drugs/alcohol concomitantly, *ICU* intensive care unit, *IQR* interquartile, *NOAF* new-onset atrial fibrillation, *SAPS* Simplified Acute Physiology Score, *SOFA* Sepsis-Related Organ Failure Assessment

^a^*p* < 0.05, when compared with the value for no arterial thromboembolic event

^b^*p* < 0.05, when compared with the value for no major bleeding event

**Table 2:** Initial transthoracic and transesophageal echocardiographic variables according to 28-day arterial thromboembolic events, major bleeding events, and death

Variable	Total (*n* = 94)	Arterial thromboembolic event		Major bleeding event		Death	
		No (*n* = 87)	Yes (*n* = 7)	No (*n* = 76)	Yes (*n* = 18)	No (*n* = 67)	Yes (*n* = 27)
LVEF, median (IQR), % (*n* = 94)	55 (45–61)	56 (45–62)	51 (33–57)	57 (49–62)	47 (43–57)	57 (49–61)	51 (30–61)^c^
LV systolic dysfunction (LVEF ≤ 45%), no. (%) (*n* = 94)	27 (29)	24 (28)	3 (43)	18 (23)	9 (50)^b^	16 (24)	11 (41)
LV systolic severe dysfunction (LVEF ≤ 30%), no. (%) (*n* = 94)	9 (10)	7 (8)	2 (29)	9 (12)	0	2 (3)	7 (26)^c^
RV dilatation, no. (%) (*n* = 90)^d^	27 (28)	25 (30)	2 (29)	22 (30)	5 (29)	21 (33)	6 (23)
Paradoxical septum, no. (%) (*n* = 94)	5 (5)	4 (5)	1 (14)	5 (5)	0	3 (4)	2 (7)
Significant left-sided valve disease, no. (%) (*n* = 94)^e^	4 (4)	2 (2)	2 (29)^a^	1 (1)	3 (17)^b^	2 (3)	2 (7)
LA area, median (IQR), cm^2^ (*n* = 70)	20 (16–24)	20 (16–23)	25 (18–28)	20 (16–23)	21 (18–24)	21 (16–25)	18 (15–22)
LA/LAA thrombus, no. (%) (*n* = 94)	0	0	0	0	0	0	0
LAA dysfunction, no. (%) (*n* = 88)^f^	17 (19)	16 (20)	1 (14)	12 (17)	5 (28)	12 (19)	5 (19)
LA/LAA dense SEC (*n* = 94)	7 (7)	6 (7)	1 (14)	6 (8)	1 (6)	5 (7)	2 (7)
LAA low velocity (*n* = 88)^g^	11 (12)	11 (14)	0	7 (10)	4 (22)	7 (11)	4 (15)
LAA emptying velocity, median (IQR), cm/s (*n* = 89)	66 (41–85)	67 (41–83)	63 (41–90)	66 (43–81)	65 (33–91)	61 (44–85)	70 (39–81)
LAA filling velocity, median (IQR), cm/s (*n* = 88)	60 (46–71)	59 (45–71)	65 (50–68)	62 (46–72)	56 (45–61)	58 (47–72)	62 (44–68)
LAA large area (> 5 cm^2^), no. (%) (*n* = 87)	13 (15)	10 (12)	3 (43)	10 (14)	3 (18)	7 (11)	6 (24)
LAA area, median (IQR), cm^2^ (*n* = 87)	3.2 (2.4–4.3)	3.2 (2.4–4.1)	4.9 (3.0–5.3)	3.2 (2.4–4.3)	3.4 (2.3–4.5)	3.4 (2.3–4.2)	3.1 (2.4–4.5)
Severe aortic atheroma, no. (%) (*n* = 91)	22 (24)	21 (25)	1 (14)	18 (24)	4 (23)	18 (27)	4 (16)

*IQR* interquartile, *LA* left atrial, *LAA* left atrial appendage, *LV* left ventricular, *LVEF* left ventricular ejection fraction, *RV* right ventricular, *SEC* spontaneous echo contrast

^a^*p* < 0.05, when compared with the value for no arterial thromboembolic event

^b^*p* < 0.05, when compared with the value for no major bleeding event

^c^*p* < 0.05, when compared with no death

^d^RV dilatation was defined as RV and LV end-diastolic areas ratio in long-axis cardiac view

^e^Aortic stenosis, *n* = 2; mitral regurgitation, *n* = 1; aortic regurgitation, *n* = 1

^f^LA/LAA dense SEC or LAA low velocity

^g^LAA emptying velocity < 25 cm/s or LAA filling velocity < 25 cm/s

### NOAF management during ICU stay

Cardioversion was attempted in 67 patients (71%), using amiodarone in 65 patients (69%) and electric shock in 23 patients (24%). Management prior to electric shock included therapeutic anticoagulation in 8 of 23 patients and TEE to exclude LA/LAA thrombus in 13 of 23 patients. During ICU stay, therapeutic anticoagulation was administered in 50 patients (53%), after 1 day (IQR: 0 to 2 days) from NOAF onset. At day 28, NOAF was persistent in 15 patients (16%) (Additional file 1: Table S4).

### Outcomes

The incidence of cardiovascular events at day 28 was 46% (95% CI: 35 to 56), and 27 patients (29%) died. 7 patients (7%) presented at least one arterial thromboembolic event occurring after a median of 6 days (IQR: 4 to 7 days) from NOAF onset. Arterial thromboembolic events included 5 ischemic strokes (three definitely, one probably, and one definitely not related to NOAF), 1 non-cerebral thromboembolism (probably related to NOAF), and 2 LAA thrombi. A second TEE was performed in 64 patients (Fig. 1) after 4.2 days (IQR: 3.3 to 5.1 days) from NOAF onset, and revealed 1 LAA thrombus. The second LAA thrombus was diagnosed in a further TEE, performed 17 days after NOAF onset because of the clinical occurrence of ischemic stroke. Details about the mechanisms of each arterial thromboembolic event are shown in Table 3. 18 patients (19%) had at least one major bleeding event occurring after a median of 8.5 days (IQR: 3.5 to 12 days) from NOAF onset. Major bleeding events included 21 extra-cranial and 2 intracranial bleedings (Additional file 1: Table S5). 10 patients (11%) presented a major bleeding event categorized as life-threatening, including fatal gastrointestinal bleeding in 2 of them. At the time of the first arterial thromboembolic and major bleeding events, therapeutic anticoagulation was used in 5 patients (71%) and 12 patients (67%), respectively.

**Table 3:** Description of 28-day arterial thromboembolic events

Variable	Patient 1	Patient 2	Patient 3	Patient 4	Patient 5	Patient 6	Patient 7
CHA_2_DS_2_-VASc score	3	6	0	1	2	5	4
NOAF characteristics	Persistent	Sustained	Sustained	Sustained	Persistent	Sustained	Persistent
Time from NOAF onset, days	11	2	6	5	5	0	3
Initial TEE study							
LV systolic function, LVEF (%)	45	50	5	56	70	60	22
LA/LAA dysfunction^a^	No	No	No	No	No	No	Yes
Severe aortic atheroma	Yes	No	No	No	No	No	No
Management before arterial thromboembolic event							
Therapeutic anticoagulation	Yes	No	Yes	Yes	Yes	No	Yes
Cardioversion attempt	Yes	Yes	Yes	Yes	No	No	No
Type	Ischemic stroke	Ischemic stroke	Ischemic stroke	Ischemic stroke	Ischemic stroke; LAA thrombus ^b^	Non-CVTE	LAA thrombus^c^
Mechanism^d^							
Causatives subtypes of ischemic stroke^e^	Probable large artery atherosclerosis	Evident cardio-aortic embolism	Evident cardio-aortic embolism	Evident cardio-aortic embolism	Evident cardio-aortic embolism	–	–
Sources of embolism other than intracardiac thrombus^f^	–	No	VA ECMO	No	No	Suspicion of endocarditis	–
Relation to NOAF	Definitely not	Definitely	Probably	Definitely	Definitely	Probably	–
Fatal ischemic stroke or non-CVTE	No	No	No	No	No	No	–

*CHA*_*2*_*DS*_*2*_*-VASc* congestive heart failure, hypertension, age ≥ 75 years (doubled), diabetes mellitus, prior stroke or transient ischemic attack or thromboembolism (doubled), vascular disease, age 65 to 74 years, sex category (female), *CVTE* cerebrovascular thromboembolism, *LA* left atrial, *LAA* left atrial appendage, *LV* left ventricular, *LVEF* left ventricular ejection fraction, *NOAF* new-onset supraventricular arrhythmia, *TEE* transesophageal echocardiography, *VA ECMO* veno-arterial extracorporeal membrane oxygenation

^a^Including LA/LAA SEC and low LAA flow velocity

^b^Revealed at a supplementary TEE performed 17 days after NOAF onset because of ischemic stroke

^c^Revealed at the second TEE study performed in all patients with arterial thromboembolic event except patient 4

^d^Arterial thromboembolic mechanism was assessed by an independent committee

^e^Causative subtype of ischemic stroke (large artery atherosclerosis, cardio-aortic embolism, small-artery occlusion, other cause, or unknown cause) was determined using the Stop Stroke Study Trial of ORG 10172 in Acute Stroke Treatment classification criteria [27]

^f^Among patients with non-cerebrovascular thromboembolism and those with evident cardio-aortic embolism as a causative subtype of ischemic stroke

### Factors associated with cardiovascular events

Baseline clinical characteristics were similar between patients with or without cardiovascular event (Additional file 1: Table S6). Among initial severity and NOAF management, septic shock and electrical cardioversion attempt on the day of NOAF onset were associated with cardiovascular events (respectively, HR: 2.72; 95% CI 1.30 to 5.69 and HR: 2.33; 95% CI 1.17 to 4.65; Additional file 1: Table S6). Regarding TEE parameters, only LV systolic dysfunction was associated with cardiovascular events (HR: 2.75; 95% CI 1.48 to 5.08; Additional file 1: Table S3). As compared with patients showing no LV systolic dysfunction, patients with mild LV systolic dysfunction had a higher cardiovascular risk (HR: 2.39; 95% CI 1.14 to 4.63) and those with severe LV systolic dysfunction had an even higher risk (HR: 4.2; 95% CI 1.79 to 10.05). A multivariable model built from TEE parameters with additional adjustment on septic shock and electrical cardioversion attempt on the day of NOAF onset identified LV systolic dysfunction (HR: 2.06; 95% CI 1.05 to 4.05) as the only independent predictor of cardiovascular events (Table 4). Additional adjustments on baseline clinical characteristics (SAPSII score, CHADS2Vasc2 and HAS-BLED) and antithrombotic management (antiplatelet therapy and therapeutic anticoagulation on the day of NOAF onset) did not substantially change the magnitude of reported hazard ratios (Additional file 1: Table S7).

**Table 4:** Univariate and multivariable analyses of factors associated with cardiovascular events^a^

Variable	Univariate analysis		Multivariable analysis	
	HR (95% CI)	*p* value	HR (95% CI)	*p* value
Septic shock^b^	2.72 (1.30–5.69)	0.01	2.36 (1.06–5.29)	0.04
Electrical cardioversion attempt^b^	2.33 (1.17–4.65)	0.02	1.51 (0.72–3.17)	0.27
LV systolic dysfunction^c, d^	2.75 (1.48–5.08)	0.001	2.06 (1.05–4.05)	0.03
LA/LAA dysfunction^d,^ ^e^	1.14 (0.54–2.39)	0.73	0.85 (0.39–1.87)	0.69
Severe aortic atheroma^d^	0.58 (0.26–1.33)	0.20	0.60 (0.26–1.40)	0.24

*CI* confidence interval, *HR* hazard ratio, *LA* left atrial, *LAA* left atrial appendage, *LV* left ventricular

^a^Composite of arterial thromboembolic events, major bleeding events, and death from any cause

^b^On the first day of new-onset atrial fibrillation onset

^c^LV ejection fraction ≤ 45%

^d^At the first echocardiography

^e^LA/LAA dense spontaneous echo contrast or LAA low velocity

### Factors associated with each of the components of cardiovascular events

#### Arterial thromboembolic event

There was no significant difference in baseline clinical characteristics, severity, and management on the day of NOAF onset between patients with and without arterial thromboembolic event, with the exception of a context of emergency surgery (Table 1). In particular, the CHA_2_DS_2_-VASc score was similar between the two groups. Regarding TEE parameters, only significant left-sided valve disease was associated with an increased risk of arterial thromboembolic event (Table 2).

#### Major bleeding event

Patients with major bleeding event more often received initial electrical cardioversion attempt, whereas the HAS-BLED score and initial therapeutic anticoagulation (Table 1) were similar between the two groups. LV systolic dysfunction and left-sided valve disease were the only TEE parameters associated with major bleeding events (Table 2).

#### 28-day mortality

Baseline clinical characteristics, initial severity and NOAF management were similar between survivors and non-survivors, with the exception of the Simplified Acute Physiology Score II score on ICU admission, which was associated with mortality (Table 1). LV ejection fraction was the only TEE parameter associated with mortality (Table 2).

## Discussion

Our study aimed at describing the early TEE abnormalities in critically ill mechanically ventilated patients with sepsis and NOAF, and at estimating the cardiovascular risk by performing a systematic comprehensive morphological work-up with TEE. We found that LV systolic dysfunction, severe aortic atheroma, and LA/LAA dysfunction were common, and that the incidence of cardiovascular events was very high, including arterial thromboembolic event (7%), major bleeding event (19%), and all-cause death (29%). Among the initial TEE abnormalities, only LV systolic dysfunction was independently associated with a poor prognosis.

### Thrombotic and bleeding risks

In line with our results, Yoshida et al. reported a 4.6% incidence of stroke in ICU patients with NOAF [28]. Walkey et al. showed that septic patients with NOAF had a greater risk of in-hospital stroke, as compared with their counterparts [2]. We also report that major bleeding events, most of which were life-threatening, were almost three times more frequent than thromboembolic events (19% versus 7%). Similarly, Walkey et al. showed a 12.6% incidence of bleeding in a large retrospective cohort of patients with sepsis and NOAF [29]. Gastrointestinal bleedings in our cohort of patients were more common and severe than in previous cohort in critically ill patients [30]. Krag et al. reported a 2.4% incidence of clinically important gastrointestinal bleeding in 1034 critically ill patients, which none of them was fatal [30]. Upper gastrointestinal lesions commonly seen in ICU patients requiring mechanical ventilation [31] may also increase bleeding risk among patients with sepsis who are receiving therapeutic anticoagulation. Therefore, thrombotic and bleeding risks in patients with sepsis and NOAF seem to be higher than those reported in cardiology wards. In a recent clinical trial studying early versus delayed cardioversion in patients with NOAF in the Emergency Department, Pluymaerkers et al. reported a 0.5% incidence of arterial thromboembolic events and no major bleeding events within 4 weeks of follow-up [32].

The underlying mechanisms of the cardiovascular events are complex in this context. NOAF may be a marker of greater sepsis severity, associated with an increased risk of thrombotic and bleeding events through hemodynamic collapse, increased systemic inflammation, and coagulopathy [33, 34]. Sepsis could predispose to LA/LAA thrombus formation in patients with NOAF. Virchow’s triad—including abnormal blood stasis, hypercoagulable state, and endothelial dysfunction—could be exacerbated in this context [35, 36]. In this setting, significant left-sided valve disease, associated with the occurrence of thrombotic events in our cohort, could be a potential etiologic factor in the development of them. In addition, due to inclusion criteria some of patients may have unrecognized pre-existing paroxysmal AF. At last, patients hospitalized in the ICU for sepsis and requiring mechanical ventilation seem to have a high baseline thrombotic and bleeding risks, irrespective of NOAF, as shown in our cohort of elderly patients with frequent comorbidities such as cardiovascular disease and arterial hypertension.

### Therapeutic anticoagulation

Observational data suggested that therapeutic anticoagulation was not associated with a reduced risk of ischemic stroke, but was associated with a higher bleeding risk among propensity score-matched patients with sepsis and atrial fibrillation [29]. However, there are no robust data on the net clinical benefit of therapeutic anticoagulation in patients with sepsis and NOAF. In line with our results, Yoshida et al. reported that 40% of critically ill patients with NOAF received therapeutic anticoagulation [28]. In patients with septic shock eligible for early NOAF cardioversion (< 24 h), a recent French survey has shown that 22% of intensivists initiated immediate therapeutic anticoagulation, whereas 27% initiated therapeutic anticoagulation after 24 h, and 44% after 48 h of sustained NOAF [37]. Similarly, we reported that only one-third of patients received therapeutic anticoagulation prior to electric shock. These deviations from recent European guidelines that recommend therapeutic anticoagulation before any emergent cardioversion [4, 10] confirm that the cardiologic approach does not seem appropriate in septic patient at high risk of bleeding. In the light of our results, specific therapeutic for NOAF in patients with sepsis such as electric shock and therapeutic anticoagulation should be used with caution. Hence, identifying patients with sepsis and NOAF most likely to benefit from therapeutic anticoagulation is a major clinical challenge. We found that the CHA_2_DS_2_-VASc and HAS-BLED scores, used for clinical decision-making on anticoagulation in the cardiology ward, were not associated with any of the cardiovascular events. Similarly, Walkey et al. reported a poor performance of CHA_2_DS_2_-VASc scores to stratify risk of stroke during sepsis in a large retrospective cohort of patients with sepsis and atrial fibrillation [29]. Finally, our results confirm the need for bedside cardiovascular risk estimation tools, to help decisions to be made about therapeutic anticoagulation and its timing.

### TEE for cardiovascular risk estimation

A TEE-guided strategy for estimating cardiovascular risk may be questionable. Although initial TEE revealed frequent LAA dysfunction (including dense SEC or low velocity), severe aortic atheroma, and LV systolic dysfunction, only the last of these was associated with a poor prognosis. Moreover, a systematic early TEE followed by a second TEE in two-thirds of patients revealed only one LAA thrombus. In line with our results, Seemann et al. reported myocardial dysfunction in 40% of patients with septic shock and NOAF [38]. LV systolic dysfunction and NOAF may be two components of myocardial septic dysfunction. The clinical and prognosis significances of LV systolic dysfunction during sepsis are a matter of debate [39]; however, our findings suggest that its association with NOAF is associated with a poor prognosis. Two previous studies reported the prevalence of thrombus, dense SEC and severe aortic atheroma in ambulatory cardiological patients undergoing TEE for NOAF cardioversion [40, 41]. In comparison with our results, Kleemann et al. reported a similar prevalence of thrombus (1%), dense SEC (9.5%), and aortic atheroma (21%), whereas Stoddart et al. reported a higher prevalence of thrombus (14%) and dense SEC (39%) [40, 41]. Those differences may be explained by the higher number of patients with structural heart disease in the study by Stoddart et al. (94%), as compared with the study by Kleemann et al. (49%) and our study (18%).

### Study strengths and limitations

This multicenter study was conducted in 10 tertiary university ICUs, where TEE is routinely used in mechanically ventilated critically ill patients. The major strengths of our study are: (i) the comprehensive search for risk factors for cardiovascular events, including systematic morphological work-up with two sequential TEE studies (initial TEE to identify patients at high risk of cardiovascular events, and the second TEE to investigate LA/LAA thrombus formation); (ii) its prospective design with adjudication of arterial thromboembolic events and major bleeding events; and (iii) the mean level of inclusion of 0.5 patient per month per center seems to be an appropriate number with regard to the eligibility criteria and is consistent with consecutive and exhaustive recruitment. Our study has several limitations. First, the relatively small number of patients limited power in all analyses. This may explain the absence of association between TEE abnormalities and outcomes. One could also object that arterial thromboembolic event might have been more relevant as a primary outcome, yet we did not consider this option, because of its low incidence. Instead, we used a composite outcome that reflects the net clinical benefit of therapeutic anticoagulation. Second, the incidence of arterial thromboembolic events may have been underestimated due to the fact that (i) the second TEE was not performed in all patients; (ii) TEEs performed in the first few days after NOAF onset may not capture LA/LAA thrombus formation within this time frame and (iii) cerebral magnetic resonance imaging was not systematic. Third, the present study was an observational study with potential indication bias. Rhythm/rate control and therapeutic anticoagulation could have influenced the occurrence of thrombotic and bleeding events. Fourth, we excluded patients who had a history of atrial fibrillation, in whom long-term cardiovascular risk stratification has been well studied [10]. Fifth, although an independent committee assessed arterial thromboembolic mechanism using a previous published classification, make a clear distinction between the “cardio-aortic embolism” and sepsis-related hypotension as a cause of stroke remains difficult. Finally, as this study was conducted in France, our findings may not be applicable elsewhere; however, the incidences of cardiovascular events reported in other nations [2, 3, 28, 29] were similar to our findings.

## Conclusions

TEE abnormalities (including LA/LAA dysfunction, severe aortic atheroma, and LV dysfunction), and cardiovascular events were common in critically ill patients with sepsis and NOAF. However, only LV systolic dysfunction was independently associated with cardiovascular events. Consequently, transesophageal echocardiography appears to be limited in this context for estimating cardiovascular risk, albeit the sample size was relatively small.

## Supplementary Information

**Additional file 1: Table S1.** Definition of CHA2DS2-VASc and HAS-BLED risk scores, arterial thromboembolic events, and bleeding events. **Table S2.** Stop Stroke Study Trial of Org 10172 in Acute Stroke Treatment (SSS-TOAST) Classification Criteria to Determine Causative Subtypes of Acute Ischemic Stroke. **Figure S1.** The decision algorithm to assign a mechanism using the Stop Stroke Study Trial of Org 10172 in Acute Stroke Treatment (SSS-TOAST) Classification Criteria. **Table S3.** Initial transthoracic and transesophageal echocardiographic variables according to 28-day cardiovascular events. **Table S4.** New onset atrial fibrillation and sepsis management during 28-day follow-up in intensive care unit. **Table S5.** Description of 28-day ISTH major bleeding events. **Table S6.** Baseline clinical characteristics at intensive care unit admission, initial severity and new-onset atrial fibrillation management according to 28-day cardiovascular events. **Table S7.** Multivariate analyses of factors associated with cardiovascular events including baseline clinical characteristics (SAPSII score, CHADS2Vasc2 and HAS-BLED) and antithrombotic management (antiplatelet therapy and therapeutic anticoagulation on the day of NOAF onset).

## Data Availability

All data and materials are fully complying with field standards and might be available after request.

## Abbreviations

CHA_2_DS_2_-VASc: Congestive heart failure hypertension, hypertension, age ≥ 75 years (doubled), diabetes mellitus, prior stroke or transient ischemic attack or thromboembolism (doubled), vascular disease, age 65 to 74 years, sex category (female)
HAS-BLED: Hypertension abnormal renal/liver function, stroke, bleeding history or predisposition, labile international normalized ratio, elderly, drugs/alcohol concomitantly
ICU: Intensive care unit
IQR: Interquartile range
LA: Left atrial
LAA: Left atrial appendage
LV: Left ventricular
NOAF: New-onset atrial fibrillation
SEC: Spontaneous echo contrast
TEE: Transesophageal echocardiography
