# Supplementary material for: Transesophageal echocardiography for cardiovascular risk estimation in patients with sepsis and new-onset atrial fibrillation: a multicenter prospective pilot study
Source: Ann Intensive Care. 2021 Oct 18;11:146. doi: 10.1186/s13613-021-00934-1 (PMC8523595; doi:10.1186/s13613-021-00934-1)
Supplement: Supplementary file 1 — Additional file 1: Table S1. Definition of CHA2DS2-VASc and HAS-BLED risk scores, arterial thromboembolic events, and bleeding events. Table S2. Stop Stroke Study Trial of Org 10172 in Acute Stroke Treatment (SSS-TOAST) Classification Criteria to Determine Causative Subtypes of Acute Ischemic Stroke. Figure S1. The decision algorithm to assign a mechanism using the Stop Stroke Study Trial of Org 10172 in Acute Stroke Treatment (SSS-TOAST) Classification Criteria. Table S3. Initial transthoracic and transesophageal echocardiographic variables according to 28-day cardiovascular events. Table S4. New onset atrial fibrillation and sepsis management during 28-day follow-up in intensive care unit. Table S5. Description of 28-day ISTH major bleeding events. Table S6. Baseline clinical characteristics at intensive care unit admission, initial severity and new-onset atrial fibrillation management according to 28-day cardiovascular events. Table S7. Multivariate analyses of factors associated with cardiovascular events including baseline clinical characteristics (SAPSII score, CHADS2Vasc2 and HAS-BLED) and antithrombotic management (antiplatelet therapy and therapeutic anticoagulation on the day of NOAF onset). [file 13613_2021_934_MOESM1_ESM.docx]

**ADDITIONNAL FILE 1**

[Table S1 Definition of CHA2DS2-VASc and HAS-BLED risk scores, arterial thromboembolic events, and bleeding events 1](#_Toc83853216)

[Table S2 Stop Stroke Study Trial of Org 10172 in Acute Stroke Treatment (SSS-TOAST) Classification Criteria to Determine Causative Subtypes of Acute Ischemic Stroke [4] 3](#_Toc83853217)

[Figure S1 The decision algorithm to assign a mechanism using the Stop Stroke Study Trial of Org 10172 in Acute Stroke Treatment (SSS-TOAST) Classification Criteria [4]. 4](#_Toc83853218)

[Table S3 Initial transthoracic and transesophageal echocardiographic variables according to 28-Day cardiovascular events 5](#_Toc83853219)

[Table S4 New onset atrial fibrillation and sepsis management during 28-Day follow-up in intensive care unit 7](#_Toc83853220)

[Table S5 Description of 28-Day ISTH major bleeding events 8](#_Toc83853221)

[Table S6 Baseline clinical characteristics at intensive care unit admission, initial severity and new onset atrial fibrillation management according to 28-Day cardiovascular events ^a^ 11](#_Toc83853222)

[Table S7 Multivariate analyses of factors associated with cardiovascular events ^a^ including baseline clinical characteristics (SAPSII score, CHADS2Vasc2 and HAS-BLED) and antithrombotic management (antiplatelet therapy and therapeutic anticoagulation on the day of NOAF onset) 13](#_Toc83853223)

| Table S1 Definition of CHA2DS2-VASc and HAS-BLED risk scores, arterial thromboembolic events, and bleeding events | |
| --- | --- |
| **Variable** | **Definition** |
| Risk score |  |
| CHA_2_DS_2_-VASc [1] | Congestive heart failure, Hypertension, Age ≥75 years (doubled), Diabetes mellitus, prior Stroke or transient ischemic attack or thromboembolism (doubled) – Vascular disease, Age 65 to 74 years, Sex category (female). |
| HAS-BLED [1] | Hypertension, Abnormal renal/liver function, Stroke, Bleeding history or predisposition, Labile international normalized ratio, Elderly, Drugs/alcohol concomitantly. |
| Stroke and non-CVTE |  |
| Stroke | An acute episode of focal or global neurological dysfunction caused by brain, spinal cord, or retinal vascular injury as a result of hemorrhage or infarction. |
| Ischemic stroke | An acute episode of focal cerebral, spinal, or retinal dysfunction caused by infarction of central nervous system tissue. Hemorrhage may be a consequence of ischemic stroke. In this situation, the stroke is an ischemic stroke with hemorrhagic transformation and not a hemorrhagic stroke. |
| Hemorrhagic stroke | An acute episode of focal, global cerebral, or spinal dysfunction caused by intraparenchymal, intraventricular, or subarachnoid hemorrhage. |
| Undetermined stroke | An acute episode of focal or global neurological dysfunction caused by presumed brain, spinal cord, or retinal vascular injury as a result of hemorrhage or infarction, but with insufficient information to allow categorization as ischemic or hemorrhagic stroke. |
| Non-CVTE | An abrupt vascular insufficiency associated with clinical or radiological evidence of arterial occlusion in the absence of other likely mechanisms. |
| Fatal stroke or non-CVTE | Death from any cause within 28 days of stroke or non-CVTE. |
| Causatives subtypes of ischemic stroke e |  |
| Bleeding | |
| Major bleeding events (ISTH definition) [2] | Meets ≥1 of the following criteria: symptomatic bleeding in a critical area or organ, e.g., intracranial, intraspinal, intraocular, retroperitoneal, intra-articular, pericardial, or intramuscular with compartment syndrome; bleeding associated with a reduction in hemoglobin of ≥ 1.24 mmol/L or leading to transfusion of ≥ 2 units blood or packed cells; fatal bleeding. |
| Life-threatening bleeding (RE-LY definition) [3] | Meets ≥1 of the following criteria (subcategory of major bleeding event): fatal bleeding; symptomatic intracranial bleeding; bleeding with a decrease in hemoglobin of ≥50 g/L, or bleeding requiring transfusion of ≥4 units of blood; necessitating surgical, endoscopic, or endovascular action. |
| Intracranial bleeding (ISTH definition) [2] | Intracerebral, subdural, epidural, or subarachnoid bleeds |
| Fatal bleeding (ISTH definition) [2] | Bleeding event that is the primary cause of death or contributes directly to death. |
| Abbreviations: CVTE, cerebrovascular thromboembolism; ISTH, International Society on Thrombosis and Haemostasis; RE-LY, Randomized Evaluation of Long-Term Anticoagulation Therapy. | |

| Table S2 Stop Stroke Study Trial of Org 10172 in Acute Stroke Treatment (SSS-TOAST) Classification Criteria to Determine Causative Subtypes of Acute Ischemic Stroke [4] | | |
| --- | --- | --- |
| **Stroke**  **Mechanism** | **Level of Confidence** | **Criteria** |
| Large artery  atherosclerosis | Evident | 1. Either occlusive or stenotic (≥50% diameter reduction) vascular disease judged to be due to atherosclerosis in the clinically relevant extracranial or intracranial  arteries, *and*  2. The absence of acute infarction in vascular territories other than the stenotic or  occluded artery |
|  | Probable | 1. Prior history of one or more transient monocular blindness (TMB), transient ischemic attacks (TIAs), or stroke from the territory of index artery affected by atherosclerosis within the last month, *or*  2. Evidence of near-occlusive stenosis or nonchronic complete occlusion judged to be due to atherosclerosis in the clinically relevant extracranial or intracranial arteries (except for the vertebral arteries), *or*  3. The presence of ipsilateral and unilateral internal watershed infarctions or multiple, temporally separate, infarctions exclusively within the territory of the affected artery |
|  | Possible | 1. The presence of an atherosclerotic plaque protruding into the lumen and causing mild stenosis (<50%) in a clinically relevant extracranial or intracranial artery and prior history of two or more TMBs, TIAs, or strokes from the territory of index artery affected by atherosclerosis, at least one event within the last month, *or*  2. Evidence for evident large artery atherosclerosis in the absence of complete diagnostic investigation for other mechanisms |
| Cardioaortic  embolism | Evident | The presence of a high-risk cardiac source of cerebral embolism (including atrial fibrillation) |
|  | Probable | 1. Evidence of systemic embolism, *or*  2. Presence of multiple acute infarctions that have occurred closely related in time within both right and left anterior or both anterior and posterior circulations in the absence of occlusion or near-occlusive stenosis of all relevant vessels; other diseases that can cause multifocal ischemic brain injury such as vasculitides, vasculopathies, and hemostatic or hemodynamic disturbances must not be present |
|  | Possible | 1. The presence of a cardiac condition with low or uncertain primary risk of cerebral embolism, *or*  2. Evidence for evident cardioaortic embolism in the absence of complete diagnostic investigation for other mechanisms |
| Small-artery  occlusion | Evident | Imaging evidence of a single clinically relevant acute infarction less than 20mm in greatest diameter within the territory of basal or brainstem penetrating arteries in the absence of any other pathology in the parent artery at the site of the origin of the penetrating artery (focal atheroma, parent vessel dissection, vasculitis, vasospasm, and so on) |
|  | Probable | The presence of stereotypic lacunar TIAs within the past week |
|  | Possible | 1. Presenting with a classical lacunar syndrome in the absence of imaging that is sensitive enough to detect small infarctions, *or*  2. Evidence for evident small artery occlusion in the absence of complete diagnostic investigation for other mechanisms |
|  | Evident | Presence of a specific disease process that involves clinically appropriate brain arteries |
|  | Probable | A specific disease process that has occurred in clear and close temporal relation to the onset of brain infarction such as arterial dissection, cardiac or arterial surgery, and cardiovascular interventions |
|  | Possible | Evidence for an evident other cause in the absence of complete diagnostic investigation for mechanisms listed above |

| Figure S1 The decision algorithm to assign a mechanism using the Stop Stroke Study Trial of Org 10172 in Acute Stroke Treatment (SSS-TOAST) Classification Criteria [4]. |
| --- |
| **** |

| Table S3 Initial transthoracic and transesophageal echocardiographic variables according to 28-Day cardiovascular events | | | | |
| --- | --- | --- | --- | --- |
| **Variable** | **Available Data** | **No Cardiovascular Events (n = 51)** | **Cardiovascular Events (n = 43)** | ***p* Value** |
| Hemodynamic parameters ^b^ |  |  |  |  |
| Time from NOAF, median (IQR), days | 93 | 1.3 (0.7–2.1) | 1.3 (0.8–2.1) | 0.48 |
| NOAF during study, No. (%) | 94 | 19 (37) | 25 (58) | 0.06 |
| Mean arterial pressure, median (IQR), mmHg | 87 | 83 (73–94) | 78 (69–86) | 0.20 |
| Heart rate, median (IQR), beats/min | 92 | 99 (82–123) | 110 (89–137) | 0.02 |
| Catecholamines, No. (%) | 93 | 29 (58) | 36 (84) | 0.03 |
| Norepinephrine | 93 | 27 (54) | 33 (78) | 0.07 |
| Epinephrine | 93 | 2 (4) | 3 (7) | 0.45 |
| Dobutamine | 93 | 1 (2) | 3 (7) | 0.29 |
| Echocardiographic parameters | | | | |
| LVEF, median (IQR), % | 94 | 58 (50–63) | 50 (36–61) | 0.002 |
| LV systolic dysfunction (LVEF ≤45%), No. (%) | 94 | 7 (14) | 20 (46) | 0.001 |
| LV systolic severe dysfunction (LVEF ≤30%), No. (%) | 94 | 1 (2) | 8 (19) | 0.004 |
| RV dilatation, No. (%) ^c^ | 90 | 18 (37) | 11 (27) | 0.50 |
| Paradoxical septum, No. (%) | 94 | 2 (4) | 3 (7) | 0.40 |
| Significant left-sided valve disease, No. (%) | 93 | 0 ( 0) | 4 ( 9) | 0.06 |
| LA area, median (IQR), cm^2^ | 70 | 20 (16–24) | 19 (16–24) | 0.88 |
| LA/LAA thrombus, No. (%) | 94 | 0 | 0 | **-** |
| LAA dysfunction ^d^, No. (%) | 88 | 7 (15) | 10 (24) | 0.73 |
| LA/LAA dense SEC | 94 | 4 (8) | 3 (7) | 0.49 |
| LAA low velocity ^e^ | 88 | 3 (6) | 8 (19) | 0.18 |
| LAA emptying velocity median (IQR), cm/s | 89 | 61 (46–82) | 69 (38–87) | 0.95 |
| LAA filling velocity, median (IQR), cm/s | 88 | 60 (48–73) | 58 (44–67) | 0.09 |
| LAA large area >5 cm^2^, No. (%) | 87 | 5 (11) | 8 (20) | 0.33 |
| LAA area, median (IQR), cm^2^ | 87 | 3.2 (2.4–4) | 3.3 (2.4–4.6) | 0.45 |
| Severe aortic atheroma, No. (%) | 91 | 15 (30) | 7 (17) | 0.20 |
| Abbreviations: IQR, interquartile; LA, left atrial; LAA, left atrial appendage; LV, left ventricular; LVEF, left ventricular ejection fraction; NOAF, new-onset atrial fibrillation; RV, right ventricular; SEC, spontaneous echo contrast; TEE, transesophageal echocardiography.  ^a^ Composite of arterial thromboembolic event, major bleeding event, or death.  ^b^ At the time of initial TEE study.  ^c^ Defined as RV and LV end-diastolic areas ratio in long-axis cardiac view >0.6.  ^d^ LA/LAA dense SEC or LAA low velocity.  ^e^ LAA emptying velocity <25 cm/s or LAA filling velocity <25 cm/s. | | | | |

| Table S4 New onset atrial fibrillation and sepsis management during 28-Day follow-up in intensive care unit | |
| --- | --- |
| **Variable** | **All Patients (n *=* 94)** |
| NOAF management during 28 days from NOAF onset |  |
| Cardioversion attempt, No. (%) | 67 (71) |
| Time from NOAF onset, median (IQR), days | 0 (0–1) |
| Medical cardioversion attempt (amiodarone), No. (%) | 65 (69) |
| Time from NOAF onset, median (IQR), days | 0 (0–1) |
| Electrical cardioversion attempt, No. (%) | 23 (24) |
| Time from NOAF onset, median (IQR), days | 1 (0–2) |
| Antiarrhythmic drug, No. (%) |  |
| Amiodarone | 77 (82) |
| Beta-blockers | 17 (18) |
| Calcium blockers | 4 (4) |
| Antiplatelet, No. (%) | 33 (35) |
| Time from NOAF onset, median (IQR), days | 0 (0–2) |
| Therapeutic anticoagulation, No. (%) *^a^* | 50 (53) |
| Time from NOAF onset, median (IQR), days | 1 (0–2) |
| Organ failure management during 28 days from NOAF onset |  |
| Catecholamines, No. (%) | 79 (84) |
| Norepinephrine | 74 (79) |
| Maximum dose, median (IQR), μg/kg/min ^b^ | 0.62 (0.40–1.65) |
| Dobutamine | 9 (10) |
| Maximum dose, median (IQR), μg/kg/min  ^b^ | 8 (5–10) |
| Epinephrine | 8 (8) |
| Maximum dose, median (IQR), μg/kg/min ^b^ | 0.76 (0.34–1.20) |
| Hemodialysis, No. (%) | 8 (8) |
| Mechanical ventilation, No. (%) | 94 (100) |
| Length of ICU stay, median (IQR), days | 1. (10–30) |
| Abbreviations: ICU, intensive care unit; IQR, interquartile; NOAF, new-onset atrial fibrillation.  ^a^ Indication (n *=* 50): NOAF (n *=* 42; 84%), extracorporeal membrane oxygenation (n *=* 2; 4%), pulmonary embolism (n *=* 4; 8%), dialysis (n *=* 1; 2%).  ^b^ Only patients who received the drug during septic shock were considered. | |

| Table S5 Description of 28-Day ISTH major bleeding events | | | | | | | | | | |
| --- | --- | --- | --- | --- | --- | --- | --- | --- | --- | --- |
| **Patient Number** ^b^ | **Age, yrs** | **HAS-BLED**  **Score** | **Septic Shock** | **Therapeutic Anticoagulation Before Bleeding** | **Time From NOAF Onset, days** ^c^ | **Source** | **Severity Variables** ^d^ | **Hemostasis Action Reported** | **Life-threatening Bleeding** ^e^ | **Fatal** |
| Patient 1 | 50 | 3 | Yes | No | 3 | Soft tissue | 1 | No | No | No |
| Patient 2 | 85 | 1 | Yes | Yes | 6 | Upper gastrointestinal | 1 | No | No | No |
|  |  |  |  | Yes | 12 | Upper gastrointestinal | 1 | No | No | No |
| Patient 3 | 69 | 4 | Yes | Yes | 19 | Soft tissue | 2 | No | Yes | No |
| Patient 4 | 40 | 2 | Yes | No | 1 | Hemoptysis | 1 | No | No | No |
|  |  |  |  | No | 6 | Upper gastrointestinal | 1 | No | No | No |
|  |  |  |  | No | 19 | Intra-articular (knee) | 0 | No | No | No |
| Patient 5 | 63 | 1 | Yes | No | 24 | Upper gastrointestinal | 1 | No | No | No |
| Patient 6 | 77 | 1 | Yes | Yes | 15 | Upper gastrointestinal | 1 | No | No | No |
|  |  |  |  | Yes | 17 | Hemoptysis | 0 | BAE | Yes | No |
| Patient 7 | 71 | 1 | Yes | No | 11 | Upper gastrointestinal | 1 | No | No | No |
| Patient 8 | 75 | 3 | Yes | Yes | 12 | Lower gastrointestinal | 2 | No | Yes | No |
|  |  |  |  | No | 28 | Lower gastrointestinal | 2 | Surgery | Yes | No |
| Patient 9 | 65 | 2 | No | Yes | 3 | Upper gastrointestinal | 2 | Surgery | Yes | Fatal |
| Patient 10 | 54 | 2 | Yes | No | 2 | Upper gastrointestinal | 2 | No | Yes | Fatal |
| Patient 11 | 69 | 2 | Yes | Yes | 5 | Upper gastrointestinal | 1 | Endoscopic hemostasis | Yes | No |
| Patient 12 | 62 | 1 | No | No | 7 | Intracranial | 0 | No | Yes | No |
| Patient 13 | 66 | 4 | Yes | Yes | 14 | Upper gastrointestinal | 2 | Endoscopic hemostasis | Yes | No |
| Patient 14 | 77 | 2 | No | Yes | 3 | Upper and lower gastrointestinal | 2 | No | Yes | No |
| Patient 15 | 61 | 1 | No | Yes | 12 | Intracranial | 0 | No | Yes | No |
| Patient 16 | 76 | 2 | No | Yes | 12 | Uterine | 1 | No | No | No |
| Patient 17 | 56 | 3 | Yes | Yes | 9 | Lower gastrointestinal | 1 | No | No | No |
| Patient 18 | 70 | 4 | Yes | Yes | 8 | Soft tissue | 1 | No | No | No |
| Abbreviations: BAE, bronchial artery embolization; HAS-BLED, Hypertension, Abnormal renal/liver function, Stroke, Bleeding history or predisposition, Labile international normalized ratio, Elderly, Drugs/alcohol concomitantly; ISTH, International Society on Thrombosis and Hemostasis; NOAF, new-onset atrial fibrillation.  ^a^ Investigator assessed.  ^b^ Three patients had two adjudicated ISTH major bleeding events and one patient had three adjudicated ISTH major bleeding events.  ^c^ 1 = day of NOAF.  ^d^ Severity variables: 1 = Bleeding associated with a reduction in haemoglobin of at least 2g/dL or leading to transfusion of two or more units of blood or packed cells; 2 = Bleeding associated with a reduction in haemoglobin of at least 5g/dL or leading to transfusion of at least four units of blood or packed cells*.*  ^e^ According to the Randomized Evaluation of Long-Term Anticoagulation Therapy (RE-LY) definition [3]. | | | | | | | | | | |

| Table S6 Baseline clinical characteristics at intensive care unit admission, initial severity and new onset atrial fibrillation management according to 28-Day cardiovascular events ^a^ | | | |
| --- | --- | --- | --- |
| **Variable** | **No Cardiovascular Event (n *=* 51)** | **Cardiovascular Event (n *=* 43)** | **p**  **Value** |
| Baseline clinical characteristics on ICU admission | | | |
| Age, IQR, interquartile, median (IQR), yrs | 69 (61–77) | 69 (62–76) | 0.83 |
| Female sex, No. (%) | 18 (35) | 15 (35) | 0.82 |
| Cardiac disease, No. (%) | 10 (20) | 7 (16) | 0.34 |
| Vascular disease, No. (%) | 7 (14) | 12 (28) | 0.20 |
| Left ventricular systolic dysfunction, No. (%) | 3 (6) | 0 | >0.99 |
| Stroke, No. (%) | 4 (8) | 4 (9) | 0.83 |
| Diabetes mellitus, No. (%) | 11 (22) | 13 (30) | 0.42 |
| Smoker, No. (%) | 29 (57) | 22 (51) | 0.64 |
| Hypertension, No. (%) | 29 (57) | 28 (65) | 0.46 |
| Clinical risk scores, median (IQR) |  |  |  |
| CHA_2_DS_2_-VASc | 3 (2–4) | 3 (2–4) | 0.61 |
| HAS-BLED | 2 (1–3) | 2 (1–3) | 0.73 |
| Previous therapeutic anticoagulation, No. (%) | 2 (4) | 2 (5) | >0.95 |
| Admission category, No. (%) |  |  |  |
| Medical | 42 (82) | 32 (75) | 0.42 |
| Scheduled surgery | 3 (6) | 2 (5) | 0.82 |
| Emergency surgery | 6 (12) | 9 (21) | 0.30 |
| Site of infection, No. (%) |  |  |  |
| Lung | 37 (72) | 31 (72) | 0.96 |
| Abdomen | 12 (23) | 10 (23) | 0.79 |
| Other | 9 (18) | 7 (16) | 0.69 |
| SAPS II score on ICU admission, median (IQR) | 54 (42–73) | 63 (52–74) | 0.18 |
| Severity and management on the day of NOAF onset | | | |
| SOFA score, median (IQR) | 9 (7–11) | 9 (6–12) | 0.79 |
| Septic shock, No. (%) | 25 (49) | 34 (79) | 0.01 |
| Catecholamines, No. (%) | 38 (74) | 37 (86) | 0.24 |
| Norepinephrine | 37 (72) | 34 (79) | 0.58 |
| Epinephrine | 1 (2) | 5 (12) | 0.03 |
| Dobutamine | 4 (8) | 3 (7) | 0.64 |
| Cardioversion attempt, No. (%) | 32 (63) | 28 (65) | 0.81 |
| Medical (amiodarone) | 31 (61) | 28 (65) | 0.67 |
| Electrical | 5 (10) | 11 (26) | 0.02 |
| Antiplatelet therapy, No. (%) | 9 (18) | 15 (35) | 0.08 |
| Therapeutic anticoagulation, No. (%) | 18 (35) | 10 (23) | 0.18 |
| Abbreviations: CHA_2_DS_2_-VASc, Congestive heart failure, Hypertension, Age ≥75 years (doubled), Diabetes mellitus, prior Stroke or transient ischemic attack or thromboembolism (doubled), Vascular disease, Age 65 to 74 years, Sex category (female); HAS-BLED, Hypertension, Abnormal renal/liver function, Stroke, Bleeding history or predisposition, Labile international normalized ratio, Elderly, Drugs/alcohol concomitantly; ICU, intensive care unit; IQR, interquartile; NOAF, new-onset atrial fibrillation; SAPS, Simplified Acute Physiology Score; SOFA, sepsis-related organ failure assessment.  ^a^ Composite of arterial thromboembolic event, major bleeding event, or death. | | | |

| Table S7 Multivariate analyses of factors associated with cardiovascular events ^a^ including baseline clinical characteristics (SAPSII score, CHADS2Vasc2 and HAS-BLED) and antithrombotic management (antiplatelet therapy and therapeutic anticoagulation on the day of NOAF onset) | | | | |
| --- | --- | --- | --- | --- |
| **Variable** |  |  | **HR (95% CI)** | **p Value** |
| CHA_2_DS_2_-VASc |  |  | 1.11 (0.91–1.35) | 0.29 |
| Septic shock ^b^ |  |  | 2.30 (1.03–5.14) | 0.04 |
| Electrical cardioversion attempt ^b^ |  |  | 1.52 (0.73–3.18) | 0.27 |
| LV systolic dysfunction ^c, d^ |  |  | 2.22 (1.12–4.40) | 0.02 |
| LA/LAA dysfunction ^d,^ ^e^ |  |  | 0.73 (0.31–1.68) | 0.46 |
| Severe aortic atheroma ^d^ |  |  | 0.61 (0.27–1.40) | 0.24 |
| HASBLED |  |  | 1.13 (0.88–1.45) | 0.33 |
| Septic shock ^b^ |  |  | 2.38 (1.06–5.32) | 0.03 |
| Electrical cardioversion attempt ^b^ |  |  | 1.45 (0.69–3.07) | 0.33 |
| LV systolic dysfunction ^c, d^ |  |  | 2.13 (1.09–4.18) | 0.03 |
| LA/LAA dysfunction ^d,^ ^e^ |  |  | 0.78 (0.35–1.75) | 0.55 |
| Severe aortic atheroma ^d^ |  |  | 0.56 (0.24–1.30) | 0.17 |
| SAPS II score |  |  | 1.00 (0.99–1.02) | > 0.99 |
| Septic shock ^b^ |  |  | 2.36 (1.04–5.34) | 0.04 |
| Electrical cardioversion attempt ^b^ |  |  | 1.51 (0.70–3.27) | 0.29 |
| LV systolic dysfunction ^c, d^ |  |  | 2.06 (1.04–4.08) | 0.04 |
| LA/LAA dysfunction ^d,^ ^e^ |  |  | 0.85 (0.39–1.88) | 0.70 |
| Severe aortic atheroma ^d^ |  |  | 0.61 (0.26–1.41) | 0.25 |
| Antiplatelet therapy ^b^ |  |  | 1.40 (0.68–2.89) | 0.37 |
| Septic shock ^b^ |  |  | 2.18 (0.95–5.00) | 0.06 |
| Electrical cardioversion attempt ^b^ |  |  | 1.62 (0.76–3.48) | 0.21 |
| LV systolic dysfunction ^c, d^ |  |  | 1.99 (1.01–3.94) | 0.05 |
| LA/LAA dysfunction ^d,^ ^e^ |  |  | 0.76 (0.33–1.74) | 0.51 |
| Severe aortic atheroma ^d^ |  |  | 0.62 (0.27–1.43) | 0.26 |
| Therapeutic anticoagulation ^b^ |  |  | 0.76 (0.36–1.62) | 0.48 |
| Septic shock ^b^ |  |  | 2.36 (1.06–5.29) | 0.04 |
| Electrical cardioversion attempt ^b^ |  |  | 1.56 (0.75–3.28) | 0.24 |
| LV systolic dysfunction ^c, d^ |  |  | 2.10 (1.07–4.11) | 0.03 |
| LA/LAA dysfunction ^d,^ ^e^ |  |  | 0.85 (0.39–1.86) | 0.68 |
| Severe aortic atheroma ^d^ |  |  | 0.63 (0.27–1.45) | 0.28 |
| Abbreviations: CI, confidence interval; HR, hazard ratio; LA, left atrial; LAA, left atrial appendage; LV, left ventricular.  ^a^ Composite of arterial thromboembolic events, major bleeding events, and death from any cause.  ^b^ On the first day of new-onset atrial fibrillation onset.  ^c^ LV ejection fraction ≤45%.  ^d^ At the first echocardiography.  ^e^ LA/LAA dense spontaneous echo contrast or LAA low velocity. | | | | |

**REFERENCES**

1. Camm AJ, Lip GYH, De Caterina R, Savelieva I, Atar D, Hohnloser SH, et al. 2012 focused update of the ESC Guidelines for the management of atrial fibrillation: an update of the 2010 ESC Guidelines for the management of atrial fibrillation. Developed with the special contribution of the European Heart Rhythm Association. Eur Heart J. 2012;33:2719–47.

2. Schulman S, Kearon C, Subcommittee on Control of Anticoagulation of the Scientific and Standardization Committee of the International Society on Thrombosis and Haemostasis. Definition of major bleeding in clinical investigations of antihemostatic medicinal products in non-surgical patients. J Thromb Haemost JTH. 2005;3:692–4.

3. Connolly SJ, Ezekowitz MD, Yusuf S, Eikelboom J, Oldgren J, Parekh A, et al. Dabigatran versus warfarin in patients with atrial fibrillation. N Engl J Med. 2009;361:1139–51.

4. Ay H, Furie KL, Singhal A, Smith WS, Sorensen AG, Koroshetz WJ. An evidence-based causative classification system for acute ischemic stroke. Ann Neurol. 2005;58:688–97.
